# Complete Genome Sequences of an *Escherichia coli* Laboratory Strain and Trimethoprim-Resistant (TMP32XR) Mutant Strains

**DOI:** 10.1128/genomeA.01434-15

**Published:** 2015-12-10

**Authors:** Abhilash Mohan, Amrisha Bhosle, Nagasuma Chandra

**Affiliations:** Department of Biochemistry, IISc, Bangalore, India

## Abstract

We report the whole-genome sequences of an *Escherichia coli* laboratory wild-type strain and trimethoprim-resistant strains (two biological replicates, TMP32XR1 and TMP32XR2). Compared to the U00096.3 strain, a widely used strain in laboratory experiments, the laboratory wild-type strain and the drug-resistant strains evolved from this (TMP32XR1 and TMP32XR2) are 13, 24, and 37 bp longer, respectively.

## GENOME ANNOUNCEMENT

*Escherichia coli* is a Gram-negative coliform bacterium, used widely as a safe laboratory model for a wide range of biological problems. At times, however, it becomes pathogenic and can cause life-threatening bloodstream infections and hard-to-treat urinary tract infections (1). Resistance in *E. coli* has been reported to be predominantly community-acquired (2). Widespread resistance to the once first-line therapeutic agent trimethoprim-sulfamethoxazole (TMP-SMX) has been a major problem, forcing the drug out of the clinic (3).The laboratory wild-type strain is sensitive to TMP and is known to acquire resistance rapidly. The laboratory wild-type *E. coli* was subjected to increasing doses of trimethoprim (TMP), and the TMP resistant strains (TMP32XR1 and TMP32XR2) were selected using standard protocols (4, 5). Two colonies at 32× the MIC for the sensitive strain were finally selected and sequenced along with the laboratory wild-type *E. coli.*

The genomes of the laboratory wild-type *E. coli* and TMP32XR1 and TMP32XR2 were sequenced using next-generation sequencing technology at Genotypic Technology Private Limited, Bangalore, India, using the Illumina HiSeq platform. A total of 13,973,607 unpaired reads for each sample with an average length of 101 bp were obtained after trimming and clipping using Trimmomatic version 0.30 (6). A template-based assembly of the genomes was performed using Bowtie2 (7). The laboratory wild-type *E. coli* was assembled using *E. coli* strain U00096.3 as the template, while TMP32XR1 and TMP32XR2 were assembled by using the wild-type *E. coli*. The draft genomes of the laboratory wild-type *E. coli* and TMP32XR1 and TMP32XR2 comprise 4,641,665 bp (4,580 genes, 4,398 coding sequences [CDSs], and 109 RNAs [87 tRNAs and 22 rRNAs]), 4,641,684 bp (4,581 genes and 4,398 CDSs), and 4,641,689 bp (4,581 genes and 4,399 CDSs), respectively.

The preprocessing of the assembled genome was performed using Picard tools version 1.119, while variant calling was performed using Genome Analysis Toolkit (GATK) version 3.1-1 (8). The GATK-prescribed best practices were followed to filter and accept single nucleotide polymorphisms (SNPs) and insertion and deletions (INDELs). The SNP and INDEL annotations for the strains were added using snpEff (9). A total of 6 INDELs and 2 SNPs were observed in the laboratory wild-type *E. coli*. TMP32XR1 showed 3 INDELs and 2 SNPs, while TMP32XR2 showed 5 INDELs and 2 SNPs.

The genome sequence of these strains will be useful for obtaining a global understanding of the mechanism of trimethoprim drug resistance in *E. coli* and may aid in the selection or development of novel drugs.

### Nucleotide sequence accession numbers.

The draft genome sequences of laboratory wild-type *E. coli* and TMP32XR1 and TMP32XR2 have been deposited at DDBJ/EMBL/GenBank under the accession numbers CP012868 (laboratory wild-type *E. coli*), CP012869 (strain TMP32XR1), and CP012870 (strain TMP32XR2).
